# Technodiscipline of Work: Does Post-Pandemic Platform Employment Generate New Psychosocial Risks?

**DOI:** 10.3390/ijerph19148609

**Published:** 2022-07-15

**Authors:** José Antonio Llosa, Esteban Agulló-Tomás

**Affiliations:** 1 Department of Social Education, Padre Ossó Faculty, University of Oviedo, 33001 Oviedo, Spain; llosajose@uniovi.es; 2 Department of Psychology, University of Oviedo, 33001 Oviedo, Spain

## 1. New Forms of Employment in the Post-Post-Academy Era, the Advance of Platform Labour

In the publication Uses of work and forms of governance: precarious work as a tool of discipline Alonso and Fernández [1] discuss a concept that is relevant in order to understand the roots of precarious work. It consists of observing the phenomenon of precarious work in disciplinary terms. These authors argue that: “in order for the worker to fulfil the company’s objectives, the need is not to supervise them, but for them to be responsible for their own supervision” [1] (p. 235). We know precarious employment as “a means for employers to shift risks and responsibilities on to workers” [2] (p. 30), a definition specified by Vosko as “one characterised by uncertainty, low pay and limited social benefits” [3] (p. 2). The conditions that we consider to be the hallmarks of precariousness are based on this conceptual corpus: temporary employment, involuntary part-time work, low pay or the deterioration of working conditions [4,5,6,7]. These elements have been deeply analysed, and their detrimental effects on welfare are widely debated [8]. Because of the conceptual breadth involved, the International Labour Organization (ILO) documentation has begun to introduce the term ‘atypical employment’ to refer to all forms of employment that are the opposite of standardised employment [9]. Standardised employment is assumed to be the one culturally desired by the population, though there are two considerations: atypical employment is so widespread and varied that it requires greater specificity in order to define the phenomena of precariousness more accurately. At present, only 25% of the world’s workers are in what could be considered standardised employment [10]. Secondly, understanding precarious employment means understanding that it has an interactive dimension with the rest of the spheres of life and thus generates precarious lives [11]. These elements come together in the idea of the discipline of precariousness, which helps to understand the neoliberal discursive dimension that legitimises employment in poor conditions: given the scarcity of employment, it begins to be seen as a privilege which, however hard the conditions, should not be given up [12].

The idea of labour discipline helps to understand the transition from stable Fordist employment to the uncertain and volatile employment of neoliberalism. The labour market’s journey through the period between the oil crisis of 1973 and the economic crisis of 2008 is a journey through the flexibilisation of labour relations, which has been legitimised normatively and scientifically, despite the negative effects on the welfare of the population [13]. It is an unfinished journey, which, through the COVID-19 pandemic, is advancing by establishing new forms of employment linked to digitalisation. Through the limitation of movement and social contact resulting from COVID-19, the so-called gig economy is experiencing global growth. This drift must be understood in regulatory terms but primarily in psychological terms. In the data, “between one to five per cent of working-age adults in the EU have, at some time, engaged in paid work mediated via an online platform” [14] (p. 16).

The gig economy is emerging as a new form of employment characteristic of the neoliberal era, based on the digital structuring of employment, which is flexible, changeable, modular, and scarcely institutionalised [15,16]. It is materialised primarily, although not exclusively, in so-called platform work: jobs deployed through a platform, where the digital platform is the only form of relationship with both clients and the company itself [15]. However, does this digitalised way of working imply a qualitatively different type of employment, does it generate new risks, or is it comparable to what we already know about precarious employment?

In formal terms, platform work, on which we will focus, is characterised by being insecure. It represents the most determined step towards the increased flexibility of labour relations, as there is no formal hiring of the people who practice it [17]. It has been understood as casual, temporary, or supplementary work, but ultimately it represents a regulatory subterfuge to create an absence of commitment between employees and employers. This quite extraordinary phenomenon is justified by questionable promises of independence and freedom for individuals or of economic expansion and dynamisation [18]. The European Commission has endorsed it: “Collaborative platforms enable individuals and other actors such as micro entrepreneurs and (small) businesses to offer services. This creates new employment, flexible working arrangements and new sources of income and helps small businesses reach a wider market and customer base. They also make markets more competitive and efficient by improving matching between demand and supply” [19] (p. 5), and the ILO points out its risks but also highlights its opportunities [20]. In this editorial, we start from the following premise: the only reason to neglect the risks that this form of employment poses to working people is the prioritisation of the interests of economic growth over the well-being of people and their labour rights.

We should point out that there is a triad of elements that define platform work: algorithmic management, the worker as a commodity and peer-to-peer (P2P).

## 2. Towards a Characterisation of Platform Work

While the definition of digitalised work involves complexity, one of the first common features we can find is peer-to-peer [21]. Through platforms, there is a direct relationship between those who consume and those who work. This aspect means direct responsibility for the employee, as the consumer is in full control of the relationship [22]. In turn, this relationship is mediated by multinational companies, such as Uber, Glovo, or Deliveroo, which provide services ranging from delivery to transport or private lessons. This characteristic generates an ambiguous line between self-employment and employed work [15]: there is no formal link between company and worker, and the risk is borne solely by the individual worker. Therefore, and going back to the initial definitions of precarity, this form of employment includes the loss of work structures and guarantees. In psychosocial terms, we would refer to two main impacts: the first has to do with risk, which in salaried work falls on the company and in this digitalised form of work falls on the employee. This logic is consistent with the flexible and precarious paradigm. The legitimisation of the European flexible employment paradigm is rooted in the assimilation of risk by employees in the face of the need to generate a labour market adaptable to global economic change [13]. However, the responsibility borne by employees in this form of employment is not accompanied by the capacity to control economic activity. Thus, it subjects people to a high level of stress due to the high level of helplessness.

Secondly, the ambiguity between salaried and employed work represents absolute uncertainty for the working person. Uncertainty about the future of work, which has a direct impact on the possibility of generating solid life projects [23], has resulting psychosocial dysfunctions. The advocates of these platform economy models present as a top priority for the viability of the model the maintenance of this flexible and adaptable capacity [13,24] without paying attention to the well-being of the people employed.

Peer-to-peer is accompanied by the second defining characteristic of the model: the worker as the exchange commodity. The meaning of work for the worker changes, as they mainly offer its availability, as an individual, to other people [25]. The focus is not so much on providing skill and competence as much as on offering as much time as possible at the direct disposal of others.

Finally, and on this feature, we will focus in more detail, algorithmic management. Defined as “mechanisms of evaluating and rewarding labour participants, evidence abounds of the role of algorithmic systems in structuring the contemporary employment economy” [26] (p. 1). Platform employees are at the disposal of an opaque algorithm that conditions the continuity and quality of the task performed. Interaction with the company they work for does not take place with another person but through a mathematical formula. This differs from traditional forms of performance evaluation since, through the algorithm, the evaluation process is intrinsically embedded in the development of the activity [27]. People are constantly evaluated, monitored, graded, and geo-localised, evaluating their performance in terms that are difficult to control. On the other hand, there is little dialectic capacity with the platform, given the absence of people in the interaction with the company for which they work.

The few existing statistics on platform work provide us with some general data. The first is that it is currently expanding globally, particularly in countries such as Spain (an estimated 18% are platform workers) or the UK [28]. Secondly, it is a form of work that is often considered to be the “future of work” [29]. The conditions of the COVID-19 pandemic have led to an expansion of these work models, which indicates that this is a perspective that is becoming established, thus the importance of legislating and analysing it in depth. The last important data available to us indicate that it is a type of employment that is mainly carried out by vulnerable groups: women, young people, and people who have been described as being difficult to employ [25]. The fact that groups that tend to be in a precarious situation are those who access these jobs is indicative of the level of risk that these forms of employment entail.

## 3. Psychosocial Risks

Although the scientific literature needs to explore these economic models in more depth, it has already been found that their associated occupational risks include a loss of job control and low autonomy [29], an increase in job insecurity [16] and a decline in employment-derived income [29]. This is related to the phenomenon of in-work poverty, defined as those people who are employed but do not earn above the poverty line. In-work poverty has been linked to a deterioration in mental health, as well as a deterioration of social relationships [30]. It is also associated with a tendency towards self-criticism [31], which is exacerbated by the increased responsibility derived from the peer-to-peer nature of platform work. It has been concluded that direct contact with clients or customers, together with the constant monitoring of activity, are features directly related to burnout [32]. Burnout implies an emotional deterioration of the employed population, together with a tendency towards the depersonalisation of the individuals, and, finally, a deterioration of mental health and health in general [33].

Beyond the impact on psychosocial well-being, these forms of work entail a decline in the capacity for trade union representation. Trade unionism has been structured around labour rights linked to the conquests materialised in labour regulations. As these forms of work are, to a large extent, alien to existing labour legislation and develop in terms of individuality, trade union organisations are facing serious difficulties in finding spaces for representation in this environment [34].

## 4. A New Psychosocial Concept: The Technodiscipline of Work

In view of the above, we consider it necessary to introduce a new concept to try to concentrate and understand this new paradigm: the technodiscipline of work.

Foucault, in Discipline and punish [35], brings back Bentham’s concept of the *panopticon* [36] to refer to explicit structures of control over life. Structures of discipline. He sees that the fundamental spaces of socialization—the company being one of them—include a system of surveillance and control, which also generate self-control. In the layout of these spaces, even in their architectural layout, but primarily in the bureaucratic sphere, there is a disciplinary system that implies a subjugation that is not explicitly perceived by the individual, insofar as it is the very individual who ends up being the architect of the subjugation. Han transposes these ideas to the digital age and introduces the notion of *psychopolitics* [37], a form of internal, psychological or psychosocial discipline. He considers that discipline today does not have a psychological impact but that its structure is fundamentally psychological and individual. Han assumes that this is the disciplinary form of neoliberalism, and we propose that algorithmic management represents the essential configuration of this scenario to give rise to what we call the technodiscipline of work.

Neoliberalism is understood as a journey towards individuality, which begins with the aim of generating a psychology of consumption and consumers, but which has an absolute social impact up to the configuration of a new individual. The neoliberal individual is at the same time interconnected and socially disconnected. Deleuze [38] thus argues that the structures of discipline of the modern world—institutions— that are closed and rigid lose their relevance, while the neoliberal discipline is based on a mobile, flexible, circumstantial, and uncertain world. The individual, unmoored, only engages in critical self-reflection, more demanding in nature than any external form of discipline. Bentham’s *panopticon* was visible and external, meaning that in neoliberal individualism we must speak of *panpsychism*, the set of psychosocial structures that delimit freedom. This idea in new management theory has been formulated to describe them as entrepreneurial subjects of themselves [39].

Thus, the relationship between the individual and the company within the context of the digital economy is not heteroarticulated or heterodetermined, but heterodetermined and self-determined at the same time. That is to say, controlled from the outside—work platforms—and internalised by the individuals themselves, who assume their rules and compete with themselves and with others. The work platform generates this effect because the counterpart of the person who works is not another individual, but a mathematical formula, an algorithm. Psychologically, mathematics is thought of as absolute, therefore unquestionable and generally indecipherable. As a result, the digital discipline of work confronts the consciousness of the worker with a mathematical examination—almost mystical—of constant evaluation [29], to which the person submits themselves, putting at stake not only the future of their work performance but time and again their worth as a worker or employee.

The final disciplining element lies in transparency and the illusion of freedom. The fact that platform work is measured in the broadest sense of the word: times, movement, scores, classification, rhythms, etc., imposes an absolute exposure to assessment. Liberal individualism cuts off social connections—the possibility of collectivising any conflict— [40], which can be seen in the practical impossibility of trade union impact in this diffuse form of employment. However, at the same time, transparent exposure suppresses the worker’s privacy and, with it, any possibility of autonomy or control over one’s own work [37]. Thus, the discourse of freedom and independence that platform work promulgates is merely aesthetic since there is no room for manoeuvre for the worker who carries out their work and has to be subjected to the dictates of the algorithm that they do not know.

As a result, the technodiscipline of work must be defined as a type of psychosocial control in which the worker’s capacity for autonomy disappears through constant digital surveillance and feedback. It generates social disengagement and individual self-questioning, which implies an absolute asymmetry of power between worker and organisation (as opposed to symmetry). Symmetry is understood as the correspondence between elements, which favours a positioning or balance as part of a whole.

The discipline of precarity is not new, and Alonso and Fernández [1] provide evidence for this. However, the fact of including algorithmic management means that the company–worker relationship is not dyadic since the worker does not have as an interlocutor anything other than a mathematical tangle that is intelligible to them. As long as there is no human counterpart in the relationship, there is no possibility of questioning the system of work organisation, and therefore only the possibility of self-questioning remains.

## 5. Conclusions: New Lines of Research

By way of conclusion, the post-pandemic labour context involves the expansion of progressively mobile, circumstantial and uncertain forms of work, which offer fewer guarantees of well-being, less capacity to develop life projects, and also less possibility of dispute in the face of limited trade union representation [34].

Phenomena such as the ‘great resignation’ in the United States and other countries meant that by December 2021, 4.3 million workers in North America voluntarily left their employment [41]. A social movement that reflects, first, the changes that are taking place in the labour market; second, the deficient conditions that these jobs are offering; third, that labour mobilisation is likely to take place in new terms and formats in the coming years; and, fourth, that one of the key elements will be the change in the meaning and paradigm of work.

In view of this, there are lines of research that are opening up or gaining momentum. The first of these has to do with the centrality of employment. In academia and social movements, employment has been idolised as a way of dignifying life [42], as well as offering quality of life. This quasi-moral understanding of employment has been a discursive element of a disciplining nature [43]. Under this historical logic, more attention has been paid to the fact of having a job than to its quality. All this at a time when the global labour market is tightening [10,12]. The second emerging line of research concerns universal basic income and its potential impact on labour dynamics. The proposal assumes that every citizen would have a monthly income sufficient for the material sustenance of life without any countervailing conditions [44]. Phenomena such as the ‘great resignation’, or the fact that it is vulnerable groups that gain access to jobs such as platform work, suggest that through the guarantee of material conditions, we would see an outright transformation of the labour market.
